# Simulation of THz Oscillations in Semiconductor Devices Based on Balance Equations

**DOI:** 10.1007/s10915-020-01311-z

**Published:** 2020-09-22

**Authors:** Tobias Linn, Kai Bittner, Hans Georg Brachtendorf, Christoph Jungemann

**Affiliations:** 1 Institute of Electromagnetic Theory, RWTH Aachen University, Kackertstr. 15-17, 52072 Aachen, Germany; 2 University of Applied Sciences of Upper Austria, 4232 Hagenberg, Austria

**Keywords:** Hyperbolic Balance Laws, THz Oscillations in Semiconductors, Well-balanced numerical Scheme, Isothermal hydrodynamic model, 65M08, 76M12, 78M12

## Abstract

Instabilities of electron plasma waves in high-mobility semiconductor devices have recently attracted a lot of attention as a possible candidate for closing the THz gap. Conventional moments-based transport models usually neglect time derivatives in the constitutive equations for vectorial quantities, resulting in parabolic systems of partial differential equations (PDE). To describe plasma waves however, such time derivatives need to be included, resulting in hyperbolic rather than parabolic systems of PDEs; thus the fundamental nature of these equation systems is changed completely. Additional nonlinear terms render the existing numerical stabilization methods for semiconductor simulation practically useless. On the other hand there are plenty of numerical methods for hyperbolic systems of PDEs in the form of conservation laws. Standard numerical schemes for conservation laws, however, are often either incapable of correctly handling the large source terms present in semiconductor devices due to built-in electric fields, or rely heavily on variable transformations which are specific to the equation system at hand (e.g. the shallow water equations), and can not be generalized easily to different equations. In this paper we develop a novel well-balanced numerical scheme for hyperbolic systems of PDEs with source terms and apply it to a simple yet non-linear electron transport model.

## Introduction

In recent years instabilities of electron plasma waves in high-electron-mobility transistors (HEMT) have attracted a lot of attention as a possible solution for closing the THz gap [4, 7, 8]. By solving the full Boltzmann Transport Equation (BTE) the behavior of such devices can be simulated in a very accurate way [13]. As transient simulations of the BTE however are computationally very expensive, especially for two or three dimensional devices, simpler transport models are needed. By using balance equations derived from the BTE together with appropriate closure relations, models of arbitrary complexity can be specified [1, 11]. The simplest of these models is the well-known Drift-Diffusion (DD) model, where only two moments of the distribution function of the BTE are considered. However, the standard DD model is not able to capture plasma wave effects, because the time derivative in the current constitutive equation is usually neglected [16]. Furthermore, two moments are not enough since heating effects of the electron gas can not be described. The closure relation is also too simplistic and inconsistent with a non-zero current density [20].

By using more balance equations as well as more advanced closure relations, better models (e.g. the full hydrodynamic model) can be developed, at the expense of higher complexity. Due to non-linear terms such models are also quite difficult to stabilize numerically, and the well-known Scharfetter-Gummel scheme [27] used for the standard DD model can not be applied anymore.

Due to the strong built-in electric fields present in semiconductor devices at the transitions between differently doped regions, the balance equations considered also contain strong and in some regions even dominating source terms, where the electron density will change by many orders of magnitude on a small length scale. Standard numerical schemes from computational fluid dynamics (CFD) are not able to capture these strong source terms in a stable way, and often lead to unphysical negative densities.

In this paper we develop a novel numerical scheme specifically for the purpose of simulating electron plasma oscillations by solving balance equations. By splitting the equations into a stationary and a dynamic part, well-balancing can be achieved even for enormous source terms, where conventional numerical schemes fail. To that end we use a one-dimensional modified hyperbolic DD model (sometimes referred to as the isothermal hydrodynamic model) including non-linearity in the flux term due to a convective derivative as a testbed for the scheme, while maintaining a simple extensibility to more advanced models including further balance equations.

## Model

**Fig. 1:**
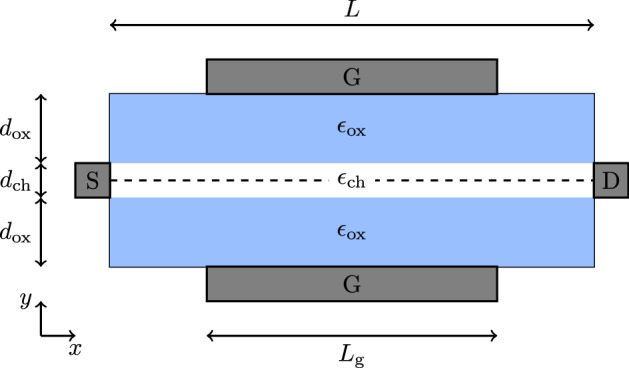
Double-Gate MOSFET with one-dimensional channel

We consider a double-gate metal-oxide-semiconductor field-effect transistor (MOSFET) with a quasi two-dimensional channel homogeneous perpendicular to the transport direction and ideal ohmic source and drain contacts shown in Fig. 1. We solve the two-dimensional Poisson equation (PE) together with a one-dimensional DD model including an additional time derivative and a convective term in the constitutive current equation. Holes can be neglected in this case. The PE is given by [28]

1 $$\begin{aligned} \varvec{\nabla }\cdot \left( \epsilon \varvec{\nabla }\varphi \right) = - e \left( N_{\mathrm {D}} - n \right) \,, \end{aligned}$$

where $\epsilon$ is the electrostatic permittivity, $\varphi$ the quasistationary potential, $e$ the elementary charge, $N_{\mathrm {D}}$ the donor concentration and $n$ is the electron density. At the contacts Dirichlet boundary conditions are used with [28]

2 $$\begin{aligned} \varphi _{\mathrm {S},\mathrm {D}}&= V_{\mathrm {S},\mathrm {D}} + V_{\mathrm {T}} {{\,\mathrm{arsinh}\,}}\left( \frac{N_\mathrm {D}}{2 n_{\mathrm {int}}} \right) \nonumber \\ \varphi _{\mathrm {G}}&= V_{\mathrm {G}} + \phi _{\mathrm {MS}}\,, \end{aligned}$$

where $V_{\mathrm {S}}, V_{\mathrm {D}}, V_{\mathrm {G}}$ are the respective source, drain and gate voltages and $V_{\mathrm {T}} = \frac{k_{\mathrm {B}} T}{e}$ is the thermal voltage, with the Boltzmann constant $k_{\mathrm {B}}$ and the temperature $T$. $\phi _{\mathrm {MS}}$ is a constant work function difference, and $n_{\mathrm {int}}$ is the intrinsic carrier density.

The DD model consists of the continuity and constitutive equation [6]

3 $$\begin{aligned} \partial _t n + \partial _x j&= 0 \end{aligned}$$

4 $$\begin{aligned} \partial _t j + \frac{j}{\tau } + \partial _x \left( c^2 n + \frac{j^2}{n} \right) + \frac{e E}{m^*} n&= 0\,, \end{aligned}$$

where $j$ is the electron current density, $c = \sqrt{\frac{k_{\mathrm {B}} T}{m^*}}$ is the thermal velocity with the effective mass $m^*$. The temperature $T$ is fixed to the lattice temperature. Furthermore, $E = - \partial _x \varphi$ is the longitudinal electric field in the channel and $\tau$ is the macroscopic relaxation time [1], which, for simplicity, is also assumed to be constant. Note that the inclusion of the convective term $\partial _x \big (\frac{j^2}{n}\big )$ leads to a non-linear equation, which complicates the numerical solution process immensely.

At the contacts we use a Dirichlet condition for the electron density (ideal ohmic contacts) [28]

5 $$\begin{aligned} n_{\mathrm {ct}} = n_{\mathrm {int}} \exp \left( {{\,\mathrm{arsinh}\,}}\left( \frac{N_{\mathrm {D}}}{2 n_{\mathrm {int}}} \right) \right) \,. \end{aligned}$$

The terminal currents including displacement currents are calculated by the Ramo-Shockley theorem [17, 26, 29].

It should be noted that this model can only serve as a rough approximation and is not sophisticated enough to model transport in nanoscale devices [23], especially in the case of high mobilities, that are necessary for plasma waves (quasi-ballistic transport). However, we chose this model because it is simple due to its inclusion of only two moments of the distribution function, while it still contains some non-linearity and can describe plasma instabilities [7]. This alleviates the development and validation of a suitable numerical solver, which can then be generalized to more advanced models as e.g. the full hydrodynamic model or other models with even higher complexity.

A critical limitation is the assumed constant thermal velocity, leading to the formation of supersonic regions in the device at sufficiently large applied voltages, when the drift velocity $v = \frac{j}{n}$ becomes larger than $c$. The transition between a sub- and a supersonic region as well as the opposite case, which leads to stationary shock-waves [21], is hard to capture in a numerical scheme. We will therefore limit our analysis to the subsonic case $|v| < c$ near equilibrium by restricting the allowed drain-source voltage to rather small values, which leads to smooth solutions [6]. This also means that the maximum voltage we can apply to savely stay in the subsonic regime is limited by the temperature through $c$.

We write eq. (3) and eq. (4) in conservation form [30]

6 $$\begin{aligned} \partial _t \varvec{U}+ \partial _x \varvec{F}(\varvec{U}) = {\mathsf {S}}\varvec{U} \end{aligned}$$

with

$$\begin{aligned} \varvec{U}= \begin{pmatrix} n \\ j \end{pmatrix} ,\quad \varvec{F}(\varvec{U}) = \begin{pmatrix} j \\ c^2 n + \frac{j^2}{n} \end{pmatrix} \quad \text {and}\quad {\mathsf {S}}= \begin{pmatrix} 0 &{} 0 \\ - \frac{e E}{m^*} &{} - \frac{1}{\tau } \end{pmatrix}\,. \end{aligned}$$

Equations of this form are frequently encountered in the field of computational fluid dynamics, for example the shallow-water equations. Due to the presence of a source term ${\mathsf {S}}\varvec{U}$ on the right-hand side, special care must be taken to achieve so called well-balancedness [12, 18]; meaning that stationary solutions, where the flux derivative is exactly canceled out by the source term, should also be exactly preserved in the discrete system. This is especially difficult in our case, because due to the large differences in the doping concentration the density can vary over many orders of magnitude in a small region, resulting in large built-in fields. A simple implementation of the finite volume scheme [10] will then result in large errors in these regions, because the flux derivative and the source term are discretized in different ways (difference of two fluxes compared to numerical integral of the source term over the cell), which can lead to numerical instability.

Since we want to perform transient simulations of plasma oscillations around a steady state, we do not need to achieve well-balancedness for all possible stationary solutions, but only for that particular steady state. To that end we split $\varvec{U}$ and ${\mathsf {S}}$ as well as $\varphi$ into a stationary and a dynamic part

7 $$\begin{aligned} \varvec{U}&= \varvec{U}^\mathrm {s}(x) + \varvec{U}^\mathrm {d}(x,t), \quad {\mathsf {S}}= {\mathsf {S}}^\mathrm {s}(x) + {\mathsf {S}}^\mathrm {d}(x,t) \nonumber \\ \varphi&= \varphi ^\mathrm {s}(x,y) + \varphi ^\mathrm {d}(x,y,t)\,, \end{aligned}$$

where $\varvec{U}^\mathrm {s}(x)$ is the solution of the stationary equation

8 $$\begin{aligned} \partial _x \varvec{F}(\varvec{U}^\mathrm {s}) = {\mathsf {S}}^\mathrm {s}\varvec{U}^\mathrm {s}\,, \end{aligned}$$

which needs to be solved prior to a transient simulation. The stationary and dynamic source matrices are given by

$$\begin{aligned} {\mathsf {S}}^\mathrm {s}= \begin{pmatrix} 0 &{} 0 \\ - \frac{e E^\mathrm {s}}{m^*} &{} - \frac{1}{\tau } \end{pmatrix} \quad \text {and}\quad {\mathsf {S}}^\mathrm {d}= \begin{pmatrix} 0 &{} 0 \\ - \frac{e E^\mathrm {d}}{m^*} &{} 0 \end{pmatrix}, \end{aligned}$$

where $E^\mathrm {s}= - \partial _x \varphi ^\mathrm {s}$ and $E^\mathrm {d}= - \partial _x \varphi ^\mathrm {d}$. The stationary and dynamic potentials both have to satisfy the corresponding version of eq. (1):

9 $$\begin{aligned} \varvec{\nabla }\cdot \left( \epsilon \varvec{\nabla }\varphi ^\mathrm {s}\right)= & {} - e \left( N_{\mathrm {D}} - n^\mathrm {s}\right) \end{aligned}$$

10 $$\begin{aligned} \varvec{\nabla }\cdot \left( \epsilon \varvec{\nabla }\varphi ^\mathrm {d}\right)= & {} e n^\mathrm {d}\,. \end{aligned}$$

In the case of the extended DD model eq. (8) leads to an ordinary differential equation for $n^\mathrm {s}$ and a constant current density $j^\mathrm {s}$

11 $$\begin{aligned} \partial _x n^\mathrm {s}&= \frac{\frac{e E^\mathrm {s}}{m^*} n^\mathrm {s}+ \frac{j^\mathrm {s}}{\tau }}{ c^2 - \left( \frac{j^\mathrm {s}}{n^\mathrm {s}}\right) ^2} \end{aligned}$$

12 $$\begin{aligned} \partial _x j^\mathrm {s}&= 0\,. \end{aligned}$$

Inserting eq. (8) into eq. (6) results in

13 $$\begin{aligned} \partial _t \varvec{U}^\mathrm {d}+ \partial _x \left( \varvec{F}(\varvec{U}) - \varvec{F}(\varvec{U}^\mathrm {s}) \right) = {\mathsf {S}}^\mathrm {s}\varvec{U}^\mathrm {d}+ {\mathsf {S}}^\mathrm {d}\varvec{U}^\mathrm {s}+ {\mathsf {S}}^\mathrm {d}\varvec{U}^\mathrm {d}\,, \end{aligned}$$

where the purely stationary contribution to the source term was replaced by a flux derivative. This modified equation can now be discretized more easily in a well-balanced manner, because the remaining source term is small near the stationary solution. Note that the last term on the right-hand side of eq. (13) is not neglected, i.e. the dynamical part is not assumed to be small compared to the stationary part.

Even though the source term in eq. (6) is linear, a similar procedure can be used if a more sophisticated model of the form

14 $$\begin{aligned} \partial _t \varvec{U}+ \partial _x \varvec{F}(\varvec{U}) = \varvec{T}(\varvec{U}, \varphi )\, \end{aligned}$$

is considered, where a non-linear source term $\varvec{T}(\varvec{U}, \varphi )$ is included. The stationary equation is then given by

15 $$\begin{aligned} \partial _x \varvec{F}(\varvec{U}^\mathrm {s}) = \varvec{T}(\varvec{U}^\mathrm {s}, \varphi ^\mathrm {s})\,. \end{aligned}$$

Subtracting eq. (15) from eq. (14) yields

16 $$\begin{aligned} \partial _t \varvec{U}^\mathrm {d}+ \partial _x \left( \varvec{F}(\varvec{U}) - \varvec{F}(\varvec{U}^\mathrm {s}) \right) = \varvec{T}(\varvec{U}^\mathrm {s}+ \varvec{U}^\mathrm {d}, \varphi ^\mathrm {s}+ \varphi ^\mathrm {d}) - \varvec{T}(\varvec{U}^\mathrm {s}, \varphi ^\mathrm {s})\,, \end{aligned}$$

corresponding to eq. (13).

## Discretization

For simplicity we use an equidistant grid in $x$ and $y$ direction with the coordinates

17 $$\begin{aligned} x_k = x_0 + k \varDelta x \quad \text {and}\quad y_l = y_0 + l \varDelta y \end{aligned}$$

for the direct and

18 $$\begin{aligned} x_{k+\frac{1}{2}} = \frac{x_k + x_{k+1}}{2} \quad \text {and}\quad y_{l+\frac{1}{2}} = \frac{y_l + y_{l+1}}{2} \end{aligned}$$

for the adjoint grid points. Note that our scheme is not fundamentally limited to the equidistant case and could easily be extended to non-equidistant grids. The PE is discretized on the 2D grid in the usual way (vertex centered finite volumes [28]), resulting in discrete values for the potential defined on the direct grid nodes.

### Stationary Problem

To discretize the stationary DD model eq. (8) we use the point-wise defined values

19 $$\begin{aligned} \varvec{U}^\mathrm {s}_{k+\frac{1}{2}} = \varvec{U}^\mathrm {s}\left( x = x_{k+\frac{1}{2}}\right) \end{aligned}$$

located at the adjoint grid points as the solution variables of the discrete equation system. Starting from an initial guess we calculate time derivatives which are then brought to zero by an iterative process.

At first the function $\varvec{U}^\mathrm {s}(x)$ is reconstructed from the solution variables by solving the stationary equation eq. (8) as an initial value problem separately in each interval starting from its midpoint and going in positive and negative $x$ direction. This computationally costly process, which can be realized by using a high-order stiff ODE solver, is necessary to enforce well-balancedness. The accuracy should be set to almost machine precision, in order to enable convergence for the whole system later. Another requirement for the ODE solver is the supply of the derivatives of the end state with respect to the initial state and the electric field, which is needed for the Newton iteration later. Here we used the fifth order RADAU IIA method described in [31].

The result is a piece-wise defined reconstruction function

20 $$\begin{aligned} \varvec{U}^\mathrm {s}(x) = \tilde{\varvec{U}}^\mathrm {s}_{k+\frac{1}{2}}(x) \quad \text {for}\quad x \in \left( x_k, x_{k+1} \right) \,, \end{aligned}$$

where

21 $$\begin{aligned} \tilde{\varvec{U}}^\mathrm {s}_{k+\frac{1}{2}}\left( x = x_{k+\frac{1}{2}}\right) = \varvec{U}^\mathrm {s}_{k+\frac{1}{2}} \end{aligned}$$

and

22 $$\begin{aligned} \partial _x \varvec{F}\left( \tilde{\varvec{U}}^\mathrm {s}_{k+\frac{1}{2}}(x)\right) = {\mathsf {S}}^\mathrm {s}_{k+\frac{1}{2}} \tilde{\varvec{U}}^\mathrm {s}_{k+\frac{1}{2}}(x)\,. \end{aligned}$$

The source matrix ${\mathsf {S}}^\mathrm {s}_{k+\frac{1}{2}}$ depends on the electric field which is calculated by finite-differences from $\varphi ^\mathrm {s}$ and is assumed to be constant in each interval $[x_k,x_{k+1}]$. Due to this fact the local solutions $\tilde{\varvec{U}}^\mathrm {s}_{k+\frac{1}{2}}(x)$ are smooth on $[x_k,x_{k+1}]$, which is important for the efficiency and accuracy of the ODE solver.

Next, the equation system including the time derivative is integrated over one interval resulting in

23 $$\begin{aligned} \int _{x_k}^{x_{k+1}} \partial _t \tilde{\varvec{U}}^\mathrm {s}_{k+\frac{1}{2}}(x) \,\mathrm {d}x + \varvec{F}(\varvec{U}^{\mathrm {s}*}_{k+1}) - \varvec{F}(\varvec{U}^{\mathrm {s}*}_{k}) = \int _{x_k}^{x_{k+1}} {\mathsf {S}}^\mathrm {s}_{k+\frac{1}{2}} \tilde{\varvec{U}}^\mathrm {s}_{k+\frac{1}{2}}(x)\, \mathrm {d}x\,, \end{aligned}$$

where the values denoted by an asterisk are located at the direct grid points between two intervals. Since there are always two values (left and right) available at each grid point, the interface states have to be calculated by a suitable averaging process. For this purpose we use an exact Riemann solver (a simple derivation can be found e.g. in [22]) which takes the full upwind information into account. The computational effort of an exact solver is quite limited in this case of only two equations, however for more advanced models an approximate solver can be used as well. The resulting upwinded interface states

24 $$\begin{aligned} \varvec{U}^{\mathrm {s}*}_{k} = \mathrm {Avg}\{\tilde{\varvec{U}}^{\mathrm {s}}_{k-\frac{1}{2}}(x_k), \tilde{\varvec{U}}^{\mathrm {s}}_{k+\frac{1}{2}}(x_k)\} \end{aligned}$$

are then shared between two adjacent intervals, ensuring charge conservation.

At the contact grid points the Dirichlet condition for the electron density leads to a semi-Riemann problem on the contact surface, since the state directly inside of the device and the density directly on the contact surface are given. A Riemann-Solver for the DD model works by connecting two initial states by two waves, where in between the two waves a constant interface state forms. In the subsonic case the two waves will travel in opposite directions. By fixing the density for the interface state to $n_{\mathrm {ct}}$, we can then calculate the missing current density on the contact surface by connecting the state on the contact to the state directly inside of the device by the respective inflowing wave. For more advanced models, more information on the contact boundaries must be supplied and more inflowing waves considered, in general one boundary condition is needed for every two balance equations.

Using the stationary reconstruction, the source term in eq. (23) can be replaced by a flux difference, resulting in

25 $$\begin{aligned} \partial _t \overline{\varvec{U}}^\mathrm {s}_{k+\frac{1}{2}} = - \frac{1}{\varDelta x} \bigg (&\varvec{F}\left( \varvec{U}^{\mathrm {s}*}_{k+1}\right) - \varvec{F}\left( \tilde{\varvec{U}}^{\mathrm {s}}_{k+\frac{1}{2}}(x_{k+1})\right) \nonumber \\&- \varvec{F}\left( \varvec{U}^{\mathrm {s}*}_{k}\right) + \varvec{F}\left( \tilde{\varvec{U}}^{\mathrm {s}}_{k+\frac{1}{2}}(x_k)\right) \bigg ) {\mathop {=}\limits ^{!}} \varvec{0}\,, \end{aligned}$$

where

26 $$\begin{aligned} \int _{x_k}^{x_{k+1}} \partial _t \tilde{\varvec{U}}^\mathrm {s}_{k+\frac{1}{2}}(x)\,\mathrm {d}x = \varDelta x \partial _t \overline{\varvec{U}}^\mathrm {s}_{k+\frac{1}{2}}\,. \end{aligned}$$

Note that even though it may be tempting to use eq. (25) for the dynamic case as well, this is not reasonable, because the reconstruction process explicitly involves solving the stationary equation for each interval. Even if we ignore the high computational cost of the ODE solver, it is not necessarily an accurate reconstruction for the dynamic case.

Self-Consistent coupling to the PE is achieved by extracting the densities at the direct grid points from the interface states and using them for the right-hand side of the PE. The stationary coupled PE-DD system is then written in residual form

27 $$\begin{aligned} \begin{pmatrix} \varvec{f}_{\varphi }^\mathrm {s}\left( \varvec{x}_{\varphi }^\mathrm {s}, \varvec{x}_{U}^\mathrm {s}\right) \\ \varvec{f}_{U}^\mathrm {s}\left( \varvec{x}_{\varphi }^\mathrm {s}, \varvec{x}_{U}^\mathrm {s}\right) \end{pmatrix} {\mathop {=}\limits ^{!}} \varvec{0}\,, \end{aligned}$$

where the residuals $\varvec{f}_{U}^\mathrm {s}$ of the DD model are the time derivatives of eq. (25). $\varvec{x}_{\varphi }^\mathrm {s}$ and $\varvec{x}_{U}^\mathrm {s}$ denote the discrete solution variables. The equation system is then solved by a damped Newton iteration, where the Jacobian is a large, sparse matrix containing the derivatives of the residuals with respect to the solution variables. These derivatives are computed as part of the solution process.

### Dynamic Problem

After solving the stationary system, its solution is fixed and we can move on to the dynamic system. As opposed to the stationary case, for the dynamic case we use the average values

28 $$\begin{aligned} \overline{\varvec{U}}^\mathrm {d}_{k} = \frac{1}{\varDelta x} \int _{x_{k-\frac{1}{2}}}^{x_{k+\frac{1}{2}}} \varvec{U}^\mathrm {d}(x)\,\mathrm {d}x \end{aligned}$$

of $\varvec{U}^\mathrm {d}$ in each vertex centered cell as the solution variables, because it leads to an easier coupling to the PE.

We use a simple piece-wise constant reconstruction of the dynamic variables in each cell. As an alternative we tested the CWENOZ3 scheme [2, 3, 19] in combination with a local characteristic decomposition [25]. Due to its third-order accuracy in smooth regions, it should be preferable to the first-order piece-wise constant reconstruction. Unfortunately, however, the resulting scheme proved to be numerically unstable in the regions with strong source terms, since the local characteristic decomposition only takes the flux Jacobian into account.

Integrating eq. (13) over one cell yields

29 $$\begin{aligned} \partial _t \overline{\varvec{U}}^\mathrm {d}_k =&- \frac{1}{\varDelta x} \bigg ( \varvec{F}\left( \varvec{U}^*_{k+\frac{1}{2}}\right) - \varvec{F}\left( \varvec{U}^\mathrm {s}_{k+\frac{1}{2}}\right) - \varvec{F}\left( \varvec{U}^*_{k-\frac{1}{2}}\right) + \varvec{F}\left( \varvec{U}^\mathrm {s}_{k-\frac{1}{2}}\right) \bigg ) \nonumber \\&+ \frac{1}{2} \left( {\mathsf {S}}^\mathrm {s}_{k-\frac{1}{2}} \overline{\varvec{U}}^{\mathrm {d}(-)}_k + {\mathsf {S}}^\mathrm {s}_{k+\frac{1}{2}} \overline{\varvec{U}}^{\mathrm {d}(+)}_k \right) + \frac{1}{2} \left( {\mathsf {S}}^\mathrm {d}_{k-\frac{1}{2}} \overline{\varvec{U}}^{(-)}_k + {\mathsf {S}}^\mathrm {d}_{k+\frac{1}{2}} \overline{\varvec{U}}^{(+)}_k \right) \,, \end{aligned}$$

where

30 $$\begin{aligned}&\overline{\varvec{U}}^{(-)}_k = \frac{2}{\varDelta x} \int _{x_{k-\frac{1}{2}}}^{x_k} \varvec{U}(x)\,\mathrm {d}x \nonumber \\ \quad \text {and}\quad&\overline{\varvec{U}}^{(+)}_k = \frac{2}{\varDelta x} \int _{x_k}^{x_{k+\frac{1}{2}}} \varvec{U}(x)\,\mathrm {d}x \end{aligned}$$

are averaged over half of a cell. For the piece-wise constant reconstruction the half cell averages are equal to the full cell averages. The variables denoted by an asterisk are as before solutions to Riemann problems, however this time located at the adjoint grid points.

At the boundaries we use the values of $\varvec{U}$ directly on the contact vertices as solution variables instead of cell averages. The reconstruction in the contact (half) cells is done by a local polynomial reconstruction satisfying

31 $$\begin{aligned} \tilde{n}^\mathrm {d}_{\mathrm {ct}}\left( x\right) |_{x_{\mathrm {ct}}}&= 0 \end{aligned}$$

32 $$\begin{aligned} \partial _x \tilde{j}^\mathrm {d}_{\mathrm {ct}}\left( x\right) |_{x_{\mathrm {ct}}}&= 0\;, \end{aligned}$$

leading to linear and quadratic reconstruction polynomials for $\tilde{n}^\mathrm {d}_{\mathrm {ct}}\left( x\right)$ and $\tilde{j}^\mathrm {d}_{\mathrm {ct}}\left( x\right)$ respectively

33 $$\begin{aligned} \tilde{n}^\mathrm {d}_{\mathrm {ct}}(x)&= \overline{n}^\mathrm {d}_{\mathrm {ct}\pm 1} \left( \pm \frac{x - x_{\mathrm {ct}}}{\varDelta x} \right) \end{aligned}$$

34 $$\begin{aligned} \tilde{j}^\mathrm {d}_{\mathrm {ct}}(x)&= j^\mathrm {d}_{\mathrm {ct}} + \left( \overline{j}^\mathrm {d}_{\mathrm {ct}\pm 1} - j^\mathrm {d}_{\mathrm {ct}} \right) \frac{12 \left( x-x_{\mathrm {ct}}\right) ^2}{13 \varDelta x^2}\,, \end{aligned}$$

where $\mathrm {ct}$ and $\mathrm {ct}\pm 1$ denote the contact and the adjacent cell.

Since the density is fixed on the contacts, its time derivative is zero. The time derivative of the current density however is not and it is obtained by evaluating eq. (13) directly on the contact, without integration over a finite volume. The spatial derivatives in that equation are evaluated consistently with the stationary and dynamic reconstructions.

The full dynamic system can then be written in the form of

35 $$\begin{aligned} \begin{pmatrix} {\mathsf {0}} &{} {\mathsf {0}} \\ {\mathsf {0}} &{} {\mathsf {D}}_U \end{pmatrix} \partial _t \begin{pmatrix} \varvec{x}_{\varphi }^\mathrm {d}\\ \varvec{x}_{U}^\mathrm {d}\end{pmatrix} + \begin{pmatrix} \varvec{f}_{\varphi }^\mathrm {d}\left( \varvec{x}_{\varphi }^\mathrm {d}, \varvec{x}_{U}^\mathrm {d}\right) \\ \varvec{f}_{U}^\mathrm {d}\left( \varvec{x}_{\varphi }^\mathrm {d}, \varvec{x}_{U}^\mathrm {d}\right) \end{pmatrix} {\mathop {=}\limits ^{!}} \varvec{0}\,, \end{aligned}$$

where ${\mathsf {D}}_U$ is a diagonal matrix. This system can then be solved by standard time-integration schemes, like the BDF methods [5] or in the frequency domain by harmonic balance [9].

## Results

**Fig. 2:**
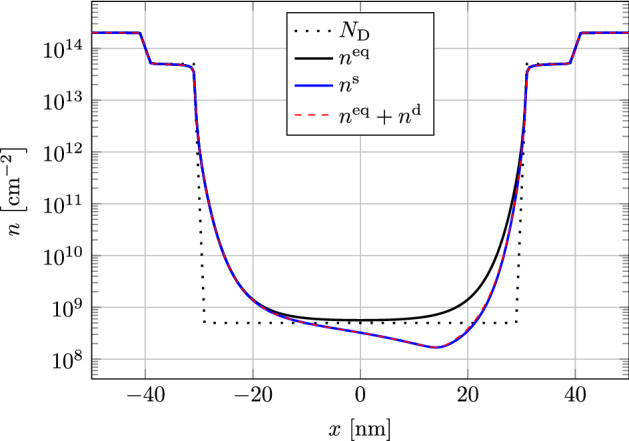
Doping profile $N_{\mathrm {D}}(x)$ and stationary electron densities for $V_{\mathrm {GS}} = {0}\hbox {V}$ and $V_{\mathrm {DS}} = {0}\hbox {V}$ ($n^{\mathrm {eq}}(x)$) and $V_{\mathrm {DS}} = {40}\hbox {mV}$ ($n^{\mathrm {s}}(x)$, $n^{\mathrm {eq}}(x) + n^{\mathrm {d}}(x)$)

We consider the device shown in Fig. 1, with a length of $L = {100}\hbox {nm}$, where the gated length is $L_{\mathrm {g}} = {60}\hbox {nm}$. The oxide and channel thicknesses are $d_{\mathrm {ox}} = {2.5}\hbox {nm}$ and $d_{\mathrm {ch}} = \hbox {nm}$ respectively. The permittivities are $\epsilon _{\mathrm {ox}} = 3.9 \, \epsilon _0$ and $\epsilon _{\mathrm {ch}} = 11.7 \, \epsilon _0$ with the vacuum permittivity $\epsilon _0$. We assume a constant relaxation time of $\tau = {0.1}{\hbox {ps}}$ and an effective mass of $m^* = 0.28621 \, m_{\mathrm {e}}$. The intrinsic carrier density is set to $n_{\mathrm {int}} = {10}^{10}\hbox { cm}^{-3}\, d_{\mathrm {ch}}$. For the potential at the gate contact we use $\phi _{\mathrm {MS}} = {0.3}{\hbox {V}}$. The simulations are performed at room temperature $T = {300}{\hbox {K}}$. The doping concentration in the channel is set to $N_{\mathrm {D}} = {10}^{15}\hbox { cm}^{-3} \, d_{\mathrm {ch}}$ while the contact regions are split into two highly doped parts with $N_{\mathrm {D}} = {10}^{20}\hbox { cm} \, d_{\mathrm {ch}}$ and $N_{\mathrm {D}} = {10}^{20}\hbox { cm}^{-3} \, d_{\mathrm {ch}}$ respectively (see Fig. 2). Between each region of constant doping an exponential transition is assumed. We use an equidistant tensor-product grid with $N_x = 201$ by $N_y = 21$ grid points.

In Fig. 2 we tested our scheme by performing stationary simulations with two different applied drain voltages. The gate voltage was set to zero $V_{\mathrm {G}} = {0}\hbox {V}$ resulting in strong built-in fields to test the numerical stability of the scheme. For equilibrium conditions, where $V_{\mathrm {S}} = V_{\mathrm {D}} = {0}\hbox {V}$, the electron density $n_{\mathrm {eq}}$ roughly follows the doping concentration. In addition to this we calculated the density for $V_{\mathrm {DS}} = {40}\hbox {mV}$ by solving the stationary equation system eq. (27) directly as well as solving the dynamic system eq. (35) for vanishing time derivatives, where the fixed stationary solution was set to the equilibrium solution. The resulting density profiles are almost identical, confirming the validity of our approach of splitting the variables into stationary and dynamic parts.

**Fig. 3:**
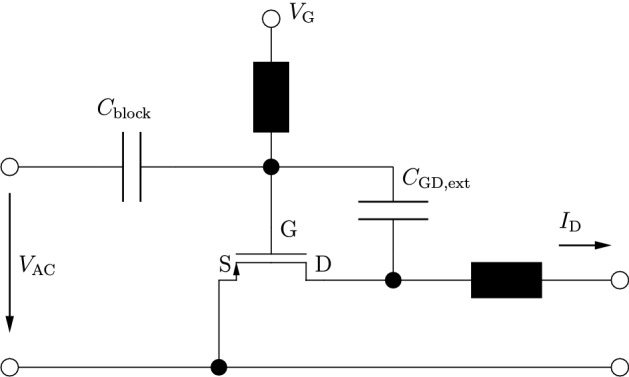
Driver circuit for resistive mixing. [15, 24]

For further analysis of our scheme we use the same driver circuit as in [15, 24], shown in Fig. 3. Due to the high admittance at THz frequencies of the coupling capacitance between the gate and drain terminal, we make the same simplification as in [14, 15] and apply the AC voltage to the source terminal instead, while keeping the gate and drain voltages constant (AC-wise grounded).

**Fig. 4:**
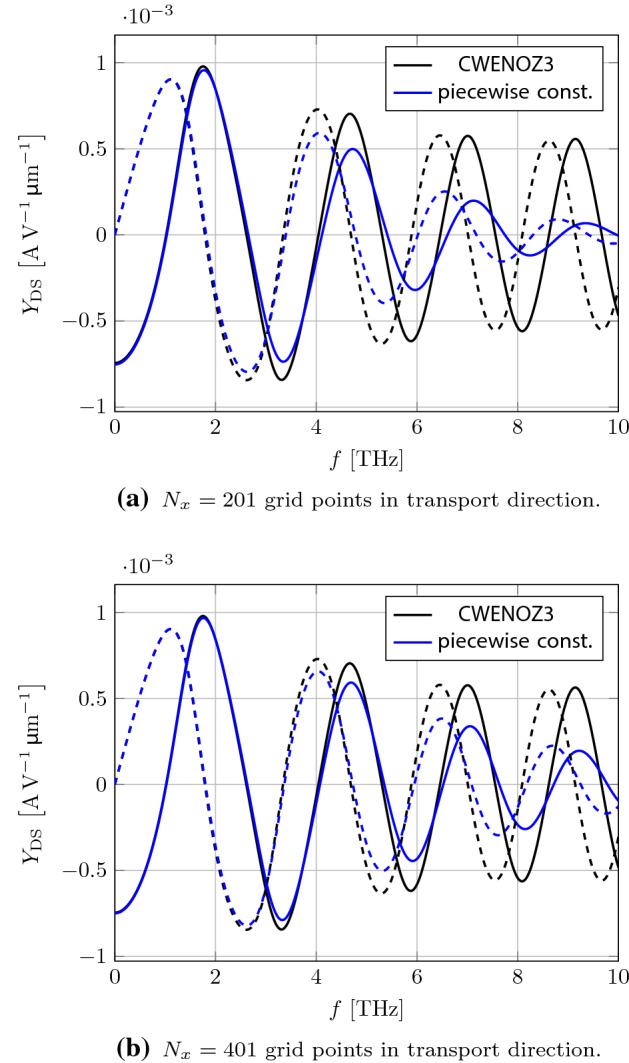
Real (solid) and imaginary (dashed) part of the drain-source admittance parameter vs the frequency for two different reconstruction methods at the DC voltage levels of $V_{\mathrm {DS}} = {0}\hbox {V}$ and $V_{\mathrm {GS}} = {0.2}\hbox {V}$

We start by calculating the small-signal drain-source admittance parameter $Y_{\mathrm {DS}}$, which is the drain current response to an AC voltage at source, where the device equations have been linearized around equilibrium with $V_{\mathrm {DS}} = 0$. To show the influence of the reconstruction procedure we compare the third-order CWENOZ3 scheme and the simple first-order piecewise constant reconstruction in Fig. 4. It is clearly visible, that the piecewise constant reconstruction introduces strong numerical damping at higher frequencies compared to the CWENOZ3 reconstruction. The stability problems we encountered for the high order reconstruction can not be seen in the small-signal regime, where the dynamic variables $\varvec{U}^\mathrm {d}$ are treated as infinitesimal quantities, leading to a perfectly smooth solution. The non-linear weights introduced in the CWENO procedure here simply reduce to the linear weights of an optimal third-order parabolic reconstruction. By increasing the number of grid points the numerical damping of the piecewise constant reconstruction can be reduced and it converges with $\mathcal {O}(\varDelta x)$ to the CWENOZ3 solution, which would only change by an negligible amount in the considered frequency region. For frequencies of up to about ${3}\hbox {THz}$ the amount of $N_{y} = 201$ grid points in transport direction chosen here seems to be sufficient to not introduce too much damping.

**Fig. 5:**
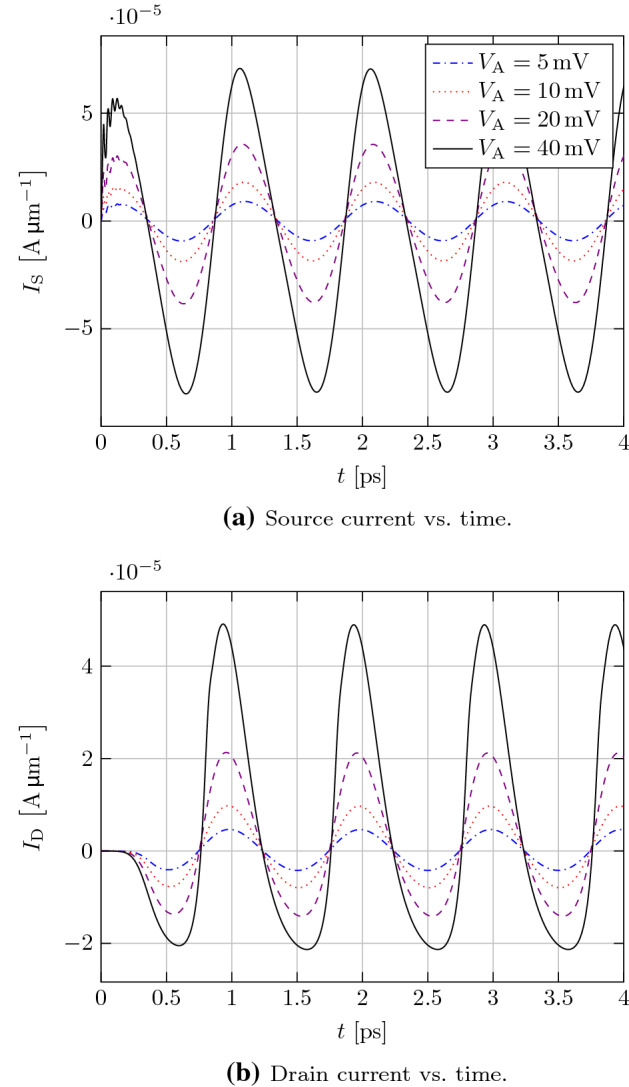
Transient source and drain terminal currents for an applied AC signal at the source terminal with different amplitudes and a frequency of $f = {1}\hbox {THz}$ at the DC voltage levels of $V_{\mathrm {DS}} = {0}\hbox {V}$ and $V_{\mathrm {GS}} = {0.2}\hbox {V}$

Going further, we performed actual transient simulations of the device by using the second-order BDF2-method [5]. The time-step size was chosen automatically by an adaptive time-step algorithm, which does not require additional information about the equation system, e.g. characteristic time scales (black-box approach). In Fig. 5 the source and drain currents are shown for different amplitudes $V_{\mathrm {A}}$ of the AC signal applied to the source terminal. After a short transient period a periodic steady-state is reached. Especially for high amplitudes the non-linear behavior of the device becomes visible in the asymmetric deformation of the drain current signal, which can not be captured by a simple small-signal analysis.

**Fig. 6:**
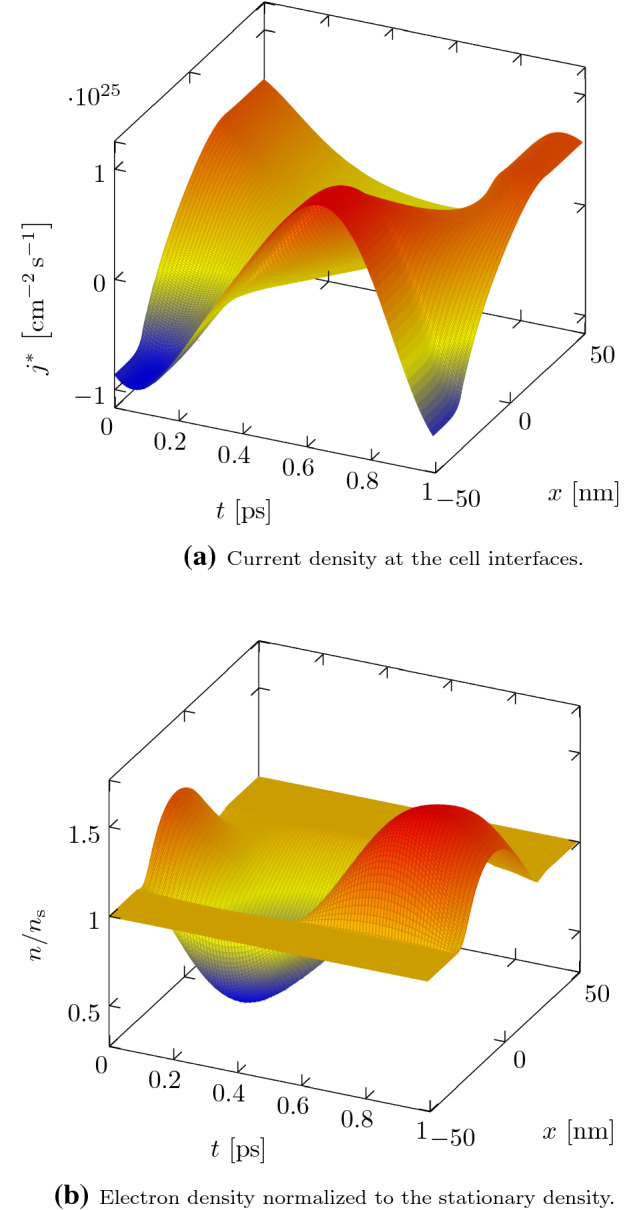
Electron and current density resolved over space and time for the periodic steady state with $V_{\mathrm {A}} = {5}\hbox {mV}$ and $f = {1}\hbox {THz}$

In Fig. 6 we show the internal variables of the periodic steady-state resolved over one period for a frequency of $f = {1}\hbox {THz}$. In Fig. 6a the current density on the cell interfaces, namely the current density component of the Riemann solutions, is shown. These current densities are also used in the calculation of the terminal currents. In the highly doped regions it is nearly constant in space, leading to a constant electron density in time. In the lowly doped channel, the propagation of waves can be observed, leading to a phase shift of the current density at the source and drain terminals, which could already be seen in the drain-source admittance parameters. In Fig. 6b the total electron density at the cell centers normalized to the stationary density is shown. As expected, in the contact regions the density is essentially constant, and only in the channel oscillations occur.

**Fig. 7:**
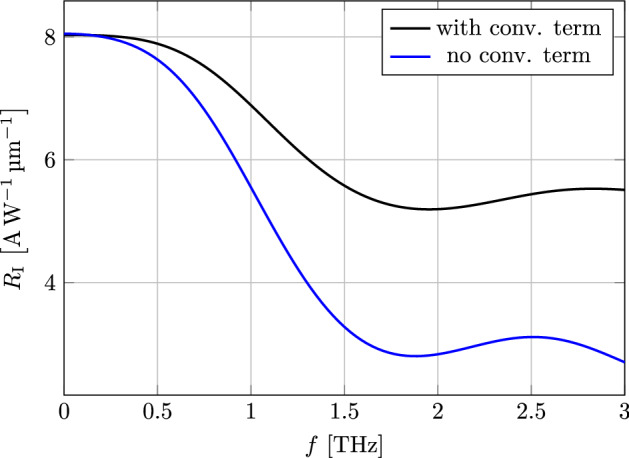
Influence of the convective derivative on the current responsivity at a DC bias of $V_{\mathrm {DS}} = {0}\hbox {V}$

We also calculated the current responsivity of the mixer given by the DC component of the current at the drain divided by the AC input power at the source terminal [15]

36 $$\begin{aligned} R_I = \frac{I_{\mathrm {D}}^{\mathrm {DC}}}{P_{\mathrm {S}}^{\mathrm {AC}}} \end{aligned}$$

with

37 $$\begin{aligned} P_{\mathrm {S}}^{\mathrm {AC}} = \frac{1}{T} \int _0^{T} V_{\mathrm {S}}(t) I_{\mathrm {S}}(t) \,\mathrm {d}t , \end{aligned}$$

averaged over one period $T$. In Fig. 7 the current responsivity at a DC bias of $V_{\mathrm {DS}} = {0}\hbox {V}$ for a frequency range of $0$ to ${3}\hbox {THz}$ is shown. To highlight the effect the convective derivative has, we repeated the calculation without it, where both of these simulations were performed using the harmonic balance approach [9]. Even though in the small-signal regime the convective derivative has no effect at equilibrium, in this case the difference is quite obvious, highlighting the nonlinear nature of the responsivity effect. For small frequencies both models yield the same result, however for larger frequencies in the THz region inclusion of the convective term leads to much larger responsivities.

## Conclusion

We developed a stable numerical method for the simulation of one dimensional balance equations with strong source terms. The equations were split into a stationary and a dynamic part, where well-balancedness was guaranteed for the stationary part. However, even for dynamic conditions different from the stationary solution, the scheme provides good results. We compared the results obtained from a low-order piecewise constant and a high order CWENOZ3 reconstruction and showed the damping effect the low-order reconstruction has at high frequencies. Furthermore, we demonstrated the capabilities of our scheme to perform nonlinear transient simulations which are able to capture important effects like the responsivity of a MOSFET mixer.

## Data Availability

The datasets generated during and/or analysed during the current study are available from the corresponding author on reasonable request.
